# Foliar application of diethyl aminoethyl hexanoate and homobrassinolide enhances cold tolerance in wheat at the jointing stage: physiological mechanisms

**DOI:** 10.3389/fpls.2026.1911607

**Published:** 2026-07-27

**Authors:** Yuanxin Shen, Junqin Yue, Ning He, Xiangdong Li, Haifang Pang, Baoting Fang, Hanfang Wang, Haiyang Jin, Deqi Zhang, Hongjian Cheng, Cheng Yang, Fei Zheng, Yunhui Shao

**Affiliations:** Wheat Research Institute, Henan Academy of Agricultural Sciences, National Engineering Laboratory of Wheat, Key Laboratory of Wheat Biology and Genetics & Breeding in Central Huanghuai Region, Ministry of Agriculture and Rural Affairs, Scientific Observation and Experimental Station of Crop Cultivation in Central Plains, Ministry of Agriculture and Rural Affairs, Henan Key Laboratory of Wheat Biology, Zhengzhou, Henan, China

**Keywords:** antioxidant enzyme activities, cellular ultrastructure, low−temperature stress, osmotic adjustment substances, plant growth regulators

## Abstract

**Introduction:**

In the Huang-Huai region of China, low spring temperatures during the jointing stage frequently cause substantial yield losses in winter wheat, posing a serious threat to wheat production.

**Methods:**

To develop a safe and effective exogenous regulation strategy, we investigated the effects of foliar application of a compound formulation containing diethyl aminoethyl hexanoate (DA-6) and 28-homobrassinolide (28-HBL) at different concentrations on two winter wheat cultivars, Bainong207 (BN207) and Zhengmai136 (ZM136), under simulated low-temperature (LT) stress (−3°C for12h) during the jointing stage. Physiological and yield-related parameters, including leaf antioxidant enzyme activities, osmoregulatory substance contents, cell ultrastructure, chlorophyll fluorescence parameters, seed-setting rates at different spikelet positions, and yield components, were evaluated to identify the optimal application rate and elucidate the physiological mechanisms underlying enhanced cold tolerance.

**Results:**

The results showed that foliar application of the DA-6/28-HBL compound formulation, particularly at 10mL/667m^2^ (N2), significantly increased the activities of leaf antioxidant enzymes, including super oxide dismutase (SOD)and catalase (CAT). It also promoted the accumulation of soluble sugars, soluble proteins, and proline while reducing malondialdehyde (MDA) content. These physiological responses effectively maintained the integrity of leaf cell ultrastructure and improved photosystemII (PSII) activity. Collectively, these improvements enhanced the cold tolerance of the plants. Compared with the water-spray treatment, the N2 treatment significantly increased the grain number per spike by 13.7% in BN207 and 14.2% in ZM136 (*p*<0.05), thereby substantially reducing yield loss under LT stress. The greatest increases in seed setting rate and grain dry weight were observed at the upper spikelet positions (G1 and G2). Among the two cultivars, BN207exhibited greater tolerance to LT stress than ZM136.

**Discussion:**

Overall, foliar application of the DA-6/28-HBL compound formulation effectively alleviated LT-induced damage during the jointing stage, providing a safe and effective exogenous strategy for enhancing cold tolerance in wheat cultivation.

## 1 Introduction

Wheat (*Triticum aestivum* L.) is one of the world’s three major cereal crops and serves as a staple food for more than 60% of the global population. Therefore, maintaining stable wheat yields is critical for ensuring global food security and reducing hunger (Shiferaw et al., 2013; FAO et al., 2024). However, climate change has increased the frequency, intensity, and duration of extreme LT events, thereby exacerbating yield instability in wheat production (Xiong et al., 2024; Augspurger, 2013; Kodra et al., 2011). This seemingly counterintuitive phenomenon occurs because global warming disrupts the stability of the polar vortex and jet stream, increasing the likelihood that cold Arctic air masses will move southward into the mid-latitudes, particularly during spring (Cohen et al., 2024; Agel et al., 2025). In recent decades, LT-induced damage has caused substantial yield losses across major wheat-producing regions worldwide, as documented in studies from the United States (Gu et al., 2008), Europe (Peings et al., 2012; Trnka et al., 2014), Australia (Ferrante et al., 2021), and China (Xiao et al., 2018; Lin et al., 2023). The Huang-Huai region is China’s most important wheat-producing area, accounting for approximately 58% of the national wheat cultivation area and 67% of total wheat production (Wang et al., 2022). However, the region is frequently affected by late spring cold events, locally known as “daochunhan,” which are among its most severe agro-meteorological hazards. These cold spells occur almost annually, with an incidence frequency of 30%–70%, typically between late March and mid-April (Zhang et al., 2021). During this period, wheat is at the jointing stage, when the developing ears are at the anther connective formation stage and are highly susceptible to low temperatures. Sudden chilling events can severely disrupt ear development, leading to a significant reduction in the seed-setting rate (Xu et al., 2022; Yu et al., 2022). Previous studies have reported that LT stress during the jointing stage can reduce grain yield per plant by 4.6%–56.4% (Wang et al., 2021). Consequently, late spring cold spells have become a major constraint to achieving high and stable wheat yields in the Huang-Huai region, highlighting the urgent need to improve cold tolerance and strengthen strategies for mitigating cold-induced damage in wheat production.

Under LT stress, the plant cell membrane system is one of the primary targets of reactive oxygen species (ROS). Excessive ROS accumulation induces membrane lipid peroxidation, leading to increased MDA content and greater membrane permeability (Ali et al., 2023). In response, plants accumulate osmoprotective substances, such as soluble sugars and proline, to maintain cellular osmotic balance while simultaneously enhancing the activities of antioxidant enzymes, including SOD, peroxidase (POD), and CAT, to scavenge excess ROS. Together, these responses help mitigate oxidative damage and protect the photosynthetic apparatus (Zulfiqar et al., 2020). As the primary site of photosynthesis, chloroplasts contain thylakoid membranes rich in unsaturated fatty acids, making them highly susceptible to oxidative stress (Ďúranová et al., 2023). Enhanced lipid peroxidation alters the physicochemical properties of chloroplast envelopes and thylakoid membranes, compromising their structural integrity and resulting in swollen, disorganized, and fewer grana lamellae. These ultrastructural changes further disrupt photosynthetic electron transport, aggravate energy metabolism imbalance, and ultimately inhibit plant growth and reduce grain yield (Soualiou et al., 2022; Liu et al., 2023). Therefore, maintaining the stability of the membrane system and preserving chloroplast structural integrity are essential for enhancing wheat tolerance to LT stress and minimizing yield losses.

The application of exogenous compounds to mitigate the adverse effects of low temperatures on wheat growth and development has become a major research focus in the development of stress-resilient wheat cultivation strategies (Dai et al., 2023; Ji et al., 2017). Plant growth regulators (PGRs) comprise a diverse group of naturally occurring and synthetic compounds that regulate plant growth, development, and stress responses. Based on their origin, PGRs are generally classified into endogenous plant hormones and synthetic plant growth regulators (Yan et al., 2024; Zahid et al., 2023). Among them, diethyl aminoethyl hexanoate (DA-6), a synthetic plant growth promoter with multiple hormone-like activities, and 28-homobrassinolide (28-HBL), a naturally occurring brassinosteroid, are recognized as safe and effective PGRs that can significantly enhance crop tolerance to cold stress (Lu et al., 2021; Sadura and Janeczko, 2022). Previous studies have demonstrated that DA-6 effectively enhances low-temperature tolerance in various crops, including kidney bean, rice, and maize seedlings, by improving photosynthetic efficiency, antioxidant capacity, and nitrogen metabolism (Bai et al., 2025; Zhang et al., 2020). Similarly, exogenous application of brassinosteroids (BRs) or their analogs has been shown to alleviate low-temperature-induced oxidative stress in a wide range of plant species by reducing ROS accumulation and membrane lipid peroxidation while simultaneously enhancing antioxidant enzyme activities and osmolyte accumulation (Gao et al., 2015; Yao et al., 2017; Sirhindi et al., 2017). Enhanced antioxidant defense appears to be a common physiological mechanism by which both DA-6 and BRs mitigate low-temperature stress, suggesting that their combined application may provide greater protection through the synergistic activation of the antioxidant system. To test this hypothesis, a preliminary column-cultivation experiment was conducted. Three days before exposure to LT stress (−3 °C for 12 h) at the jointing stage, two wheat cultivars were sprayed with water (CK), DA-6 (26.6 mg/L), 28-HBL (0.033 mg/L), and a combination of the two. The results showed that the combined treatment significantly increased the number of kernels per spike and thousand-kernel weight, resulting in higher grain yield per column and reduced yield loss (**Supplementary Table 1**). These findings confirmed the synergistic effect of DA-6 and 28-HBL in wheat, consistent with previous results reported in rice (Wu, 2019). However, the physiological effects of compound formulations are generally concentration-dependent, and their efficacy varies with application rate (Dai et al., 2025).

Previous studies have mainly focused on the effects of individual plant growth regulators on wheat yield and antioxidant physiology, whereas studies on the combined application of DA-6 and 28-HBL under low-temperature stress during the wheat jointing stage remain limited, particularly those integrating antioxidant physiological responses with chloroplast ultrastructural observations. Furthermore, the optimal application concentration of this compound formulation and the physiological mechanisms underlying its enhancement of cold tolerance in wheat have not yet been elucidated. To address these knowledge gaps, the present study employed a column-cultivation system under simulated LT stress conditions (−3 °C for 12 h) during the wheat jointing stage. Three days before LT treatment, wheat plants were foliar-sprayed with different concentrations of the DA-6/28-HBL compound formulation. Following stress exposure, physiological and yield-related parameters were evaluated, including leaf antioxidant enzyme activities, osmolyte contents, cellular ultrastructure, photosynthetic characteristics, seed-setting rates at different spikelet positions, and yield components at maturity. The objectives of this study were to determine the optimal application concentration of the DA-6/28-HBL formulation and to elucidate the physiological and structural mechanisms by which it enhances cold tolerance in wheat under LT stress at the jointing stage through the coordinated regulation of antioxidant enzyme activities, osmolyte accumulation, and chloroplast structural stability. The findings of this study are expected to provide both a theoretical basis and practical guidance for the development of cultivation strategies aimed at enhancing cold tolerance in wheat.

## 2 Materials and methods

### 2.1 Experimental site and design

The experiment was conducted during the 2024–2025 growing season at the Modern Agricultural Research and Development Base in Henan Province (Pingyuan New District, Xinxiang City; 35°00′N, 113°40′E). A column-cultivation system was used, with soil collected from the 0–30 cm plough layer of a local field. The soil was classified as loam and had the following properties: organic matter, 11.58 g/kg; total nitrogen, 1.18 g/kg; available nitrogen, 78.4 mg/kg; available phosphorus, 9.0 mg/kg; and available potassium, 98.8 mg/kg. After sieving, the soil was packed into cylindrical columns (21 cm in diameter × 60 cm in height), with the bottoms covered by mesh screens. After compaction, the columns were buried in the field with the soil surface inside each column level with the surrounding ground. Two widely cultivated winter wheat (*Triticum aestivum* L.) cultivars in the Huang-Huai region, Bainong 207 (BN207) and Zhengmai 136 (ZM136), were used in this study (**Supplementary Table 2**). Seeds were sown on October 26, 2024, with each cultivar planted in 150 columns, each containing approximately 15 plants. At the three-leaf stage, seedlings were thinned to 10 plants per column. Throughout the growing season, all field management practices followed standard high-yield cultivation protocols. The monthly average rainfall and temperature during the experimental period are shown in **Supplementary Figure 1**.

After the wheat plants reached the stamen and pistil differentiation stage, young ears from the main stems were sampled daily at 08:00 and examined under a stereomicroscope. Foliar spray treatments were applied before 09:00 on the day the young ears reached the anther connective formation stage. Four treatments were established: CK (plants sprayed with an equivalent volume of water before LT treatment); N1 (plants sprayed with the DA-6/28-HBL compound formulation at 5 mL/667 m^2^ before LT treatment; the formulation contained 7.99% DA-6 and 0.01% 28-HBL, resulting in final concentrations of 13.3 mg/L DA-6 and 0.017 mg/L 28-HBL, based on a spray volume of 30 L/667 m^2^); N2 (plants sprayed with the DA-6/28-HBL compound formulation at 10 mL/667 m^2^ before LT treatment, resulting in final concentrations of 26.6 mg/L DA-6 and 0.033 mg/L 28-HBL); and N3 (plants sprayed with the DA-6/28-HBL compound formulation at 15 mL/667 m^2^ before LT treatment, resulting in final concentrations of 39.9 mg/L DA-6 and 0.050 mg/L 28-HBL). All treatments were applied as a fine mist until the leaf surfaces were uniformly wetted without runoff.

Three days after foliar application, according to previous methods and with slight modifications, the plants were subjected to artificial LT stress in a climate-controlled chamber (Wang et al., 2024b; Chen et al., 2026). After transfer to the chamber, the temperature was gradually reduced from ambient temperature to −3 °C at a rate of 0.5 °C min^-1^. Once the target temperature was reached and stabilized, the plants were exposed to −3 °C for 12 h under 70% relative humidity in complete darkness (0 lux). The chamber temperature reached −3 °C before 20:00 on March 28, and the 12-h LT treatment was maintained until 08:00 on March 29. After the treatment, the columns were returned to their original field positions and reburied, allowing the plants to continue growing under natural field conditions until maturity.

### 2.2 Sampling and measurements

#### 2.2.1 Determination of leaf antioxidant enzyme activities and osmolyte contents

On the day following the LT treatment, three wheat columns were randomly selected from each treatment. From each column, three uniformly growing plants were chosen, and the uppermost fully expanded leaf from each plant was collected. The leaf samples were immediately wrapped in aluminum foil, flash-frozen in liquid nitrogen, and stored at −80 °C in an ultra-low-temperature freezer for subsequent determination of antioxidant enzyme activities and osmolyte contents. Before analysis, the frozen leaf samples were placed in a mortar, flash-frozen with liquid nitrogen, and rapidly ground into a fine powder using a pestle. SOD activity was determined using the nitroblue tetrazolium (NBT) method (Ma et al., 2021), CAT activity was measured using the ammonium molybdate colorimetric method (An et al., 2024), and MDA content was determined using the thiobarbituric acid (TBA) method (Abdel-Kader et al., 2018).

All assays for antioxidant enzymes and MDA were performed using a microplate reader and commercial assay kits (SOD, CAT, and MDA detection kits; Suzhou Comin Biotechnology Co., Ltd., China) according to the manufacturer’s instructions. Briefly, 0.1 g of leaf tissue was homogenized in 1 mL of extraction buffer in an ice bath, and the homogenate was centrifuged at 8,000 × *g* for 10 min at 4 °C. The resulting supernatant was transferred to a 96-well microplate and mixed with the appropriate assay reagents following the prescribed protocols. Absorbance was measured using a microplate reader at the specified wavelengths: 560 nm for SOD activity, where A560(CK) and A560(B) represent the absorbance of the control and sample wells, respectively; 405 nm for CAT activity, where ΔA denotes the change in total absorbance during the reaction period; and 532 nm and 600 nm for MDA content, corresponding to A532 and A600, respectively. The activities of SOD and CAT, as well as the MDA content, were calculated using the equations provided in the assay kit manuals (Equations 1–3, respectively). Each treatment consisted of three biological replicates for the determination of SOD activity, CAT activity, and MDA content.

(1) $$\text{SOD Activity }(\text{U}/\text{g Fresh Weight})=\;\frac{20\times ({\text{A}}_{560}(\text{CK})-{\text{A}}_{560}(\text{B}))}{{\text{A}}_{560}(\text{B})\times \text{W}}$$

(2) $$\text{CAT Activity }(\text{U}/\text{g Fresh Weight})=\frac{8.9\times (\Delta \text{A}-0.0013)}{\text{W}}$$

(3) $$\text{MDA Content }(\text{nmol}/\text{g Fresh Weight})=\;\frac{51.6\times ({\text{A}}_{532}-{\text{A}}_{600})}{\text{W}}$$

W: is the mass of the sample (g).

Proline content was determined using the sulfosalicylic acid method described by Bates et al. (1973), which is widely used to quantify proline accumulation in plants under stress conditions. Briefly, 0.5 g of leaf tissue (three biological replicates per treatment) was homogenized in 5 mL of 3% (w/v) sulfosalicylic acid and heated in a boiling water bath for 10 min. After cooling, the homogenate was centrifuged, and 2 mL of the supernatant was collected. Subsequently, 2 mL of glacial acetic acid and 2 mL of 2.5% (w/v) acid ninhydrin solution were added, and the mixture was thoroughly vortexed. The reaction mixture was then heated in a boiling water bath for 60 min to allow color development, cooled to room temperature, and extracted with 5 mL of toluene. After vortexing and phase separation, the toluene phase was collected, and its absorbance was measured at 520 nm. A standard curve was prepared using a series of proline standard solutions, and the proline content of the samples was calculated accordingly. Proline content was expressed as μg/g fresh weight (FW). The reported values were used for comparative analysis among treatments and do not represent absolute proline concentrations.

Soluble sugar content was determined using the anthrone–sulfuric acid method, with a standard curve prepared from a series of sucrose standard solutions of known concentrations (Zhao et al., 2021). Briefly, 0.2 g of fresh leaf tissue was placed in a test tube containing 5 mL of deionized water and heated in a boiling water bath for 30 min. After cooling and filtration, the extract was diluted with 10 mL of deionized water. Subsequently, 0.5 mL of the diluted extract was mixed with 1.5 mL of deionized water, 0.5 mL of 2% (w/v) anthrone reagent, and 5 mL of concentrated sulfuric acid. The mixture was allowed to develop color before absorbance was measured at 630 nm using a spectrophotometer. Soluble sugar content was then calculated from the sucrose standard curve.

Soluble protein content was determined using the Coomassie Brilliant Blue G-250 dye-binding method (Cheng et al., 2024). Briefly, 250 μL of the enzyme extract was mixed with 2.5 mL of Coomassie Brilliant Blue G-250 reagent and incubated at room temperature for 2 min. The absorbance was then measured at 595 nm, and the soluble protein content was calculated using a standard curve prepared with known concentrations of bovine serum albumin (BSA).

#### 2.2.2 Observation of leaf cellular ultrastructure

On the day following LT treatment, leaf sections (approximately 1 mm²) were excised from the middle region of the apical leaves on the main stems, excluding the midrib, and immediately immersed in 2.5% glutaraldehyde fixative (pH 7.2) in medical vials. Air was carefully removed from the samples using a syringe to ensure complete infiltration of the fixative. The samples were then fixed at 4 °C for 4 h. After primary fixation, the samples were rinsed three times (15 min per rinse) with 0.1 M phosphate buffer (PB, pH 7.4). They were subsequently post-fixed in 1% osmium tetroxide prepared in 0.1 M PB (pH 7.4) for 7 h at room temperature in the dark. The samples were subsequently rinsed three times with phosphate buffer, dehydrated through a graded ethanol series, and embedded in Epon 812 resin to obtain polymerized blocks. The embedded blocks were sectioned into ultrathin sections (60 nm) using a Leica UC7 ultramicrotome. The sections were then stained with 2.6% lead citrate in a carbon dioxide-free environment and examined using a transmission electron microscope (HT7800/HT7700, Japan). For each treatment, three ultrathin sections were randomly selected for observation (Semenova et al., 2017).

#### 2.2.3 Determination of photosynthetic characteristics

Following LT treatment, photosynthetic characteristics were measured at 3-day intervals. Relative chlorophyll content was determined using a SPAD-502 chlorophyll meter (Minolta, Japan) and expressed as SPAD values. For each treatment, the uppermost fully expanded leaves were selected for measurement, with flag leaves used after their emergence. One SPAD reading was taken at the widest part of each leaf, and the mean value of 10 leaves was calculated to represent the relative chlorophyll content.

Chlorophyll fluorescence parameters were measured between 09:00 and 11:00 using an FMS 2 pulse-modulated fluorometer (Hansatech Instruments Ltd., Norfolk, UK). Before measurement, leaves were dark-adapted for 30 min. The minimum chlorophyll fluorescence yield (Fo) was then recorded with the photosystem II (PSII) reaction centers fully open, followed by measurement of the maximum chlorophyll fluorescence yield (Fm) under a saturating light pulse of 8,000 μmol photons m^-2^ s^-1^ with a pulse duration of 0.7 s, during which the PSII reaction centers were fully closed. The maximum photochemical efficiency of PSII was calculated as Fv/Fm = (Fm – Fo)/Fm. The leaves were then exposed to actinic light at 1,200 μmol photons m^-2^ s^-1^ for 5 min until the fluorescence signal reached a steady state. Steady-state fluorescence (Fs) was recorded, after which the same saturating light pulse was applied to determine the maximum fluorescence under light-adapted conditions (Fm′). The effective photochemical efficiency of PSII (*ΦPSII*) was calculated as *ΦPSII* = (Fm′ – Fs)/Fm′. For each treatment, measurements were taken from the uppermost 10 fully expanded leaves, and the results are presented as the mean of these measurements.

#### 2.2.4 Determination of grain-setting rate and grain weight at different spikelet positions

At maturity, three wheat columns with uniform plant growth were randomly selected from each treatment. The main stem spikes were harvested, and the spikelets on each spike were numbered sequentially along the rachis from the base to the apex. Following the method of Chen et al. (2024), each spike was divided into three sections: the lower section, consisting of the basal six spikelets; the upper section, consisting of the apical six spikelets; and the middle section, comprising the remaining spikelets. Individual grain positions within each spikelet were designated as G1, G2, G3, and G4 (Xu et al., 2021b). The dry weight of grains at each position was measured using an analytical balance with a precision of 0.0001 g.

#### 2.2.5 Determination of grain yield and yield components

At maturity, three wheat columns were randomly selected from each treatment as biological replicates to determine the number of spikes per plant, grains per spike, 1000-grain weight, and grain yield per column.

### 2.3 Data analysis

Statistical analyses were performed using SPSS version 26.0 (IBM Corp., Armonk, NY, USA). Two-way analysis of variance (ANOVA), with cultivar and treatment as fixed factors, was used to evaluate the main effects and their interaction. One-way ANOVA was then conducted to assess differences among treatments within each cultivar. When significant differences were detected (p < 0.05), mean comparisons were performed using Duncan’s multiple range test. All figures were prepared using Origin 2021 (OriginLab Corp., Northampton, MA, USA).

## 3 Results

### 3.1 Effects of the DA-6/28-HBL compound formulation on leaf antioxidant activity and osmoregulatory substances in wheat under LT stress at the jointing stage

**Figure 1:**
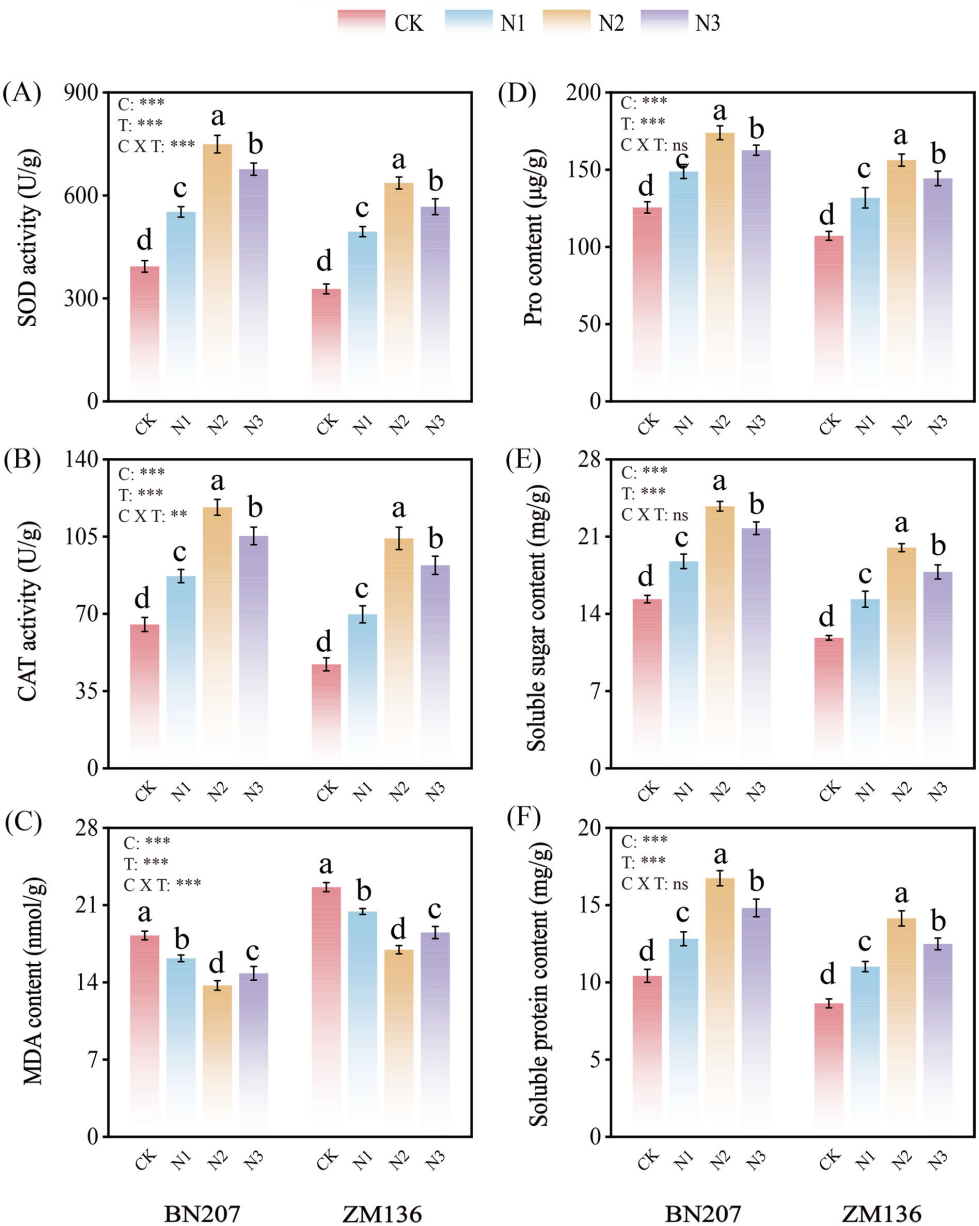
Effects of different concentrations of the DA-6/28-HBL compound formulation on leaf antioxidant enzyme activities and osmolyte contents in two wheat cultivars. A–C: Effects on antioxidant enzyme activities: **(A)** superoxide dismutase (SOD) activity, **(B)** catalase (CAT) activity, and **(C)** malondialdehyde (MDA) content. **(D–F)**: Effects on osmolyte contents: **(D)** proline, **(E)** soluble sugars, and **(F)** soluble proteins. BN207, Bainong 207; ZM136, Zhengmai 136; C, Cultivar; T, Treatment; CK, water spray; N1, 5 mL/667 m^2^; N2, 10 mL/667 m^2^; N3, 15 mL/667 m^2^. Data are presented as mean ± SD (n = 3). Different lowercase letters indicate significant differences among treatments within each cultivar according to Duncan’s multiple range test at *p* < 0.05. *F*-values followed by **, and *** represent significance at *p* <0.01, and *p* <0.001, respectively; ns indicates no significant effect. Data are presented on a fresh weight basis.

As illustrated in **Figure 1**, foliar application of the DA-6/28-HBL compound formulation prior to LT stress significantly enhanced antioxidant enzyme activities and the accumulation of osmotic adjustment substances in the leaves of both wheat cultivars. Compared with the CK treatment, all three application rates (N1–N3) significantly increased SOD and CAT activities and promoted the accumulation of proline, soluble sugars, and soluble proteins, while simultaneously reducing MDA content (*p* < 0.05). Among the treatments, N2 showed the most pronounced effects. In BN207, N2 significantly increased SOD and CAT activities by 90.6% and 81.5%, respectively, relative to CK, while MDA content decreased by 24.8% (*p* < 0.05). In addition, proline, soluble sugars, and soluble protein contents increased by 38.5%, 54.8%, and 60.7%, respectively.

A similar trend was observed in ZM136. Compared with the CK treatment, N2 significantly increased SOD and CAT activities by 94.1% and 121.2%, respectively. In addition, proline, soluble sugar, and soluble protein contents increased significantly by 45.7%, 69.1%, and 63.7%, respectively, while MDA content decreased significantly by 25.1% (*p* < 0.05). These results indicate that N2 was the most effective treatment for enhancing antioxidant enzyme activity and promoting the accumulation of osmoprotective substances, thereby alleviating membrane lipid peroxidation and improving plant tolerance to LT stress.

Under the same treatment conditions, comparison between the two cultivars showed that BN207 exhibited higher antioxidant enzyme activities, greater accumulation of osmotic adjustment substances, and lower MDA content than ZM136. These results indicate that BN207 has a stronger inherent capacity for cold tolerance.

### 3.2 Effects of the DA-6/28-HBL compound formulation on the cellular ultrastructure of wheat functional leaves under LT stress at the jointing stage

**Figure 2:**
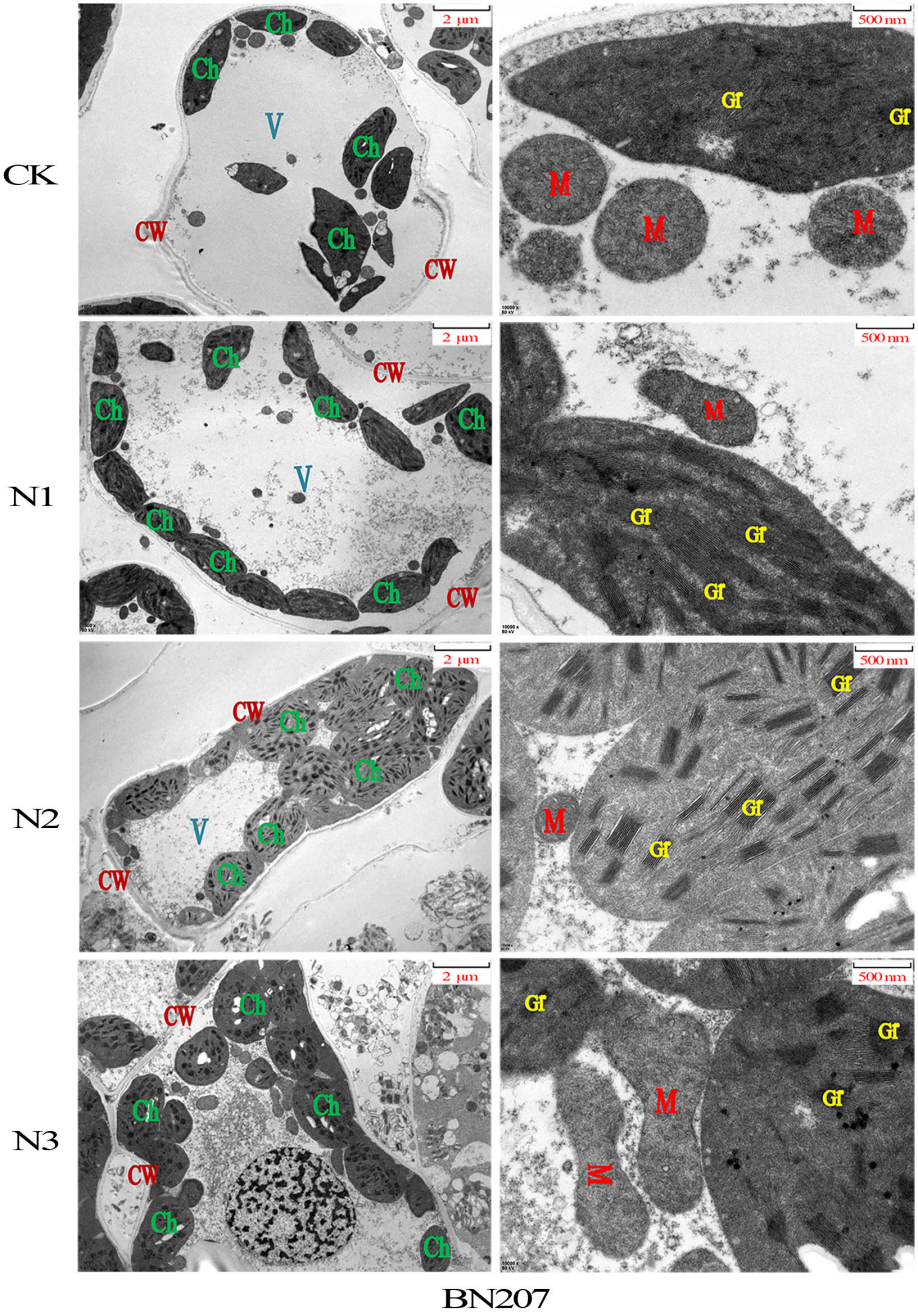
Effects of different concentrations of the DA-6/28-HBL compound formulation on the cellular ultrastructure of functional leaves in BN207. CW, cell wall; Ch, chloroplast; Gr, grana; M, mitochondria; V, vacuole; C, Cultivar; T, Treatment; BN207, Bainong 207; CK, water spray; N1, 5 mL/667 m^2^; N2, 10 mL/667 m^2^; N3, 15 mL/667 m^2^.

**Figure 3:**
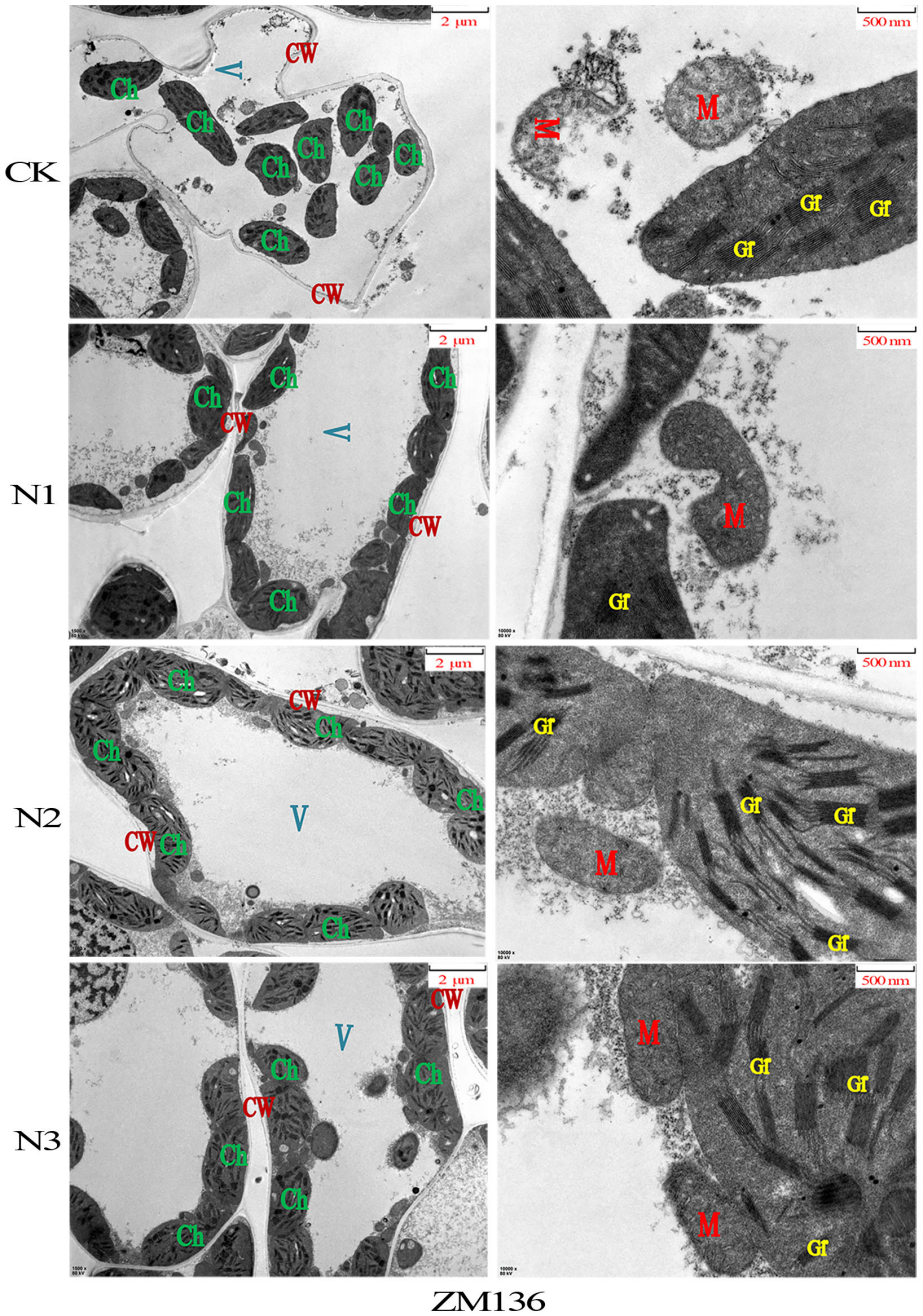
Effects of different concentrations of the DA-6/28-HBL compound formulation on the cellular ultrastructure of functional leaves in ZM136. CW, cell wall; Ch, chloroplast; Gr, grana; M, mitochondrion; V, vacuole; C, Cultivar; T, Treatment; ZM136, Zhengmai 136; CK, water spray; N1, 5 mL/667 m^2^; N2, 10 mL/667 m^2^; N3, 15 mL/667 m^2^.

Under CK treatment, chloroplast distribution was altered in both wheat cultivars. In addition, mitochondria in BN207 leaves showed partial disorganization (**Figure 2**). In contrast, mitochondria in ZM136 leaves were more severely affected, appearing irregularly arranged, with some exhibiting structural damage, indistinct double membranes, and reduced matrix electron density (**Figure 3**). These observations indicate that LT stress directly damages the cellular ultrastructure of wheat leaves under the CK treatment, with ZM136 exhibiting more severe structural injury than BN207.

As the concentration of the DA-6/28-HBL compound formulation increased, leaf cellular structure progressively recovered. Under the N1 treatment, only a few mitochondria and individual chloroplasts were irregularly arranged in both cultivars. In contrast, under the N2 and N3 treatments, mesophyll cells of both cultivars exhibited intact cell walls and numerous chloroplasts, which were predominantly arranged along the cell periphery. Within these chloroplasts, grana thylakoids were tightly and orderly stacked, aligned along the longitudinal axis of the chloroplasts, with well-preserved structures and fewer osmiophilic granules. Compared with the N3 treatment, N2 resulted in more defined mitochondrial double membranes, better-preserved cristae, and more distinct vacuoles. These results demonstrate that foliar application of the DA-6/28-HBL compound formulation prior to LT stress significantly alleviated damage to leaf cellular ultrastructure. Among all treatments, N2 exhibited the strongest protective effect, resulting in the least ultrastructural damage.

In the functional leaves of both wheat cultivars, chloroplast number, chloroplast area, and chloroplast length-to-width ratio initially increased with increasing treatment concentration and then declined at higher concentrations, reaching their maximum under the N2 treatment. Significant differences were observed among treatments (**Table 1**). Compared with the CK treatment, N2 significantly increased chloroplast number, chloroplast area, and chloroplast length-to-width ratio in BN207 by 70.5%, 65.4%, and 39.1%, respectively. In ZM136, the corresponding increases were 76.0%, 85.2%, and 43.8%, respectively (*p* < 0.05). These findings indicate that, under LT stress, the N2 treatment was most effective in maintaining chloroplast structural integrity in wheat leaves.

**Table 1:** Effects of different concentrations of the DA-6/28-HBL compound formulation on chloroplast structural parameters in the functional leaves of two wheat cultivars.

Cultivar	Treatment	Nr. of chloroplasts	Chloroplast area (μm2)	Chloroplast length/width
BN207	CK	10 d	13.62 d	1.79 d
	N1	13 c	17.19 c	2.08 c
	N2	18 a	22.53 a	2.49 a
	N3	16 b	20.83 b	2.38 b
ZM136	CK	8 d	9.59 d	1.53 d
	N1	10 c	12.61 c	1.79 c
	N2	14 a	17.61 a	2.20 a
	N3	13 b	15.74 b	2.06 b
F value	Cultivar	493.7***	3769.5***	864.3***
	Treatment	512.3***	2444.5***	940.8***
	Cultivar X Treatment	7.4***	9.7***	1.6ns

Data are presented as the mean (n = 3). Different lowercase letters indicate significant differences among treatments within each cultivar according to Duncan’s multiple range test at *p* < 0.05. *F*-values followed by *, **, and *** indicate significance at *p* < 0.05, *p* < 0.01, and *p* < 0.001, respectively; ns indicates no significant effect. BN207, Bainong 207; ZM136, Zhengmai 136; CK, water spray; N1, 5 mL/667 m^2^; N2, 10 mL/667 m^2^; N3, 15 mL/667 m^2^.

The responses of thylakoid system parameters to different concentrations of the compound formulation mirrored the trends observed for chloroplast structural parameters in both wheat cultivars. As shown in **Table 2**, all measured thylakoid parameters increased progressively with increasing treatment concentration, reaching their maximum under the N2 treatment, and then declined significantly at higher concentrations. Significant differences were observed among treatments, and all treated samples showed significantly higher values than the CK.

**Table 2:** Effects of different concentrations of the DA-6/28-HBL compound formulation on thylakoid system parameters in the functional leaves of two wheat cultivars.

Cultivar	Treatment	Nr. of grana	Nr. of lamellae	Base thickness
				(μm)
BN207	CK	17 d	15 d	0.17 c
	N1	20 c	18 c	0.21 b
	N2	26 a	23 a	0.26 a
	N3	25 b	22 b	0.24 a
ZM136	CK	13 d	11 d	0.14 c
	N1	17 c	14 c	0.17 b
	N2	22 a	19 a	0.23 a
	N3	21 b	18 b	0.21 a
F value	Cultivar	531.6***	975.0***	9.5**
	Treatment	659.6***	812.5***	18.1***
	Cultivar X Treatment	0.5ns	1.5ns	1.8ns

Data are presented as the mean (n = 3). Different lowercase letters indicate significant differences among treatments within each cultivar according to Duncan’s multiple range test at *p* < 0.05. The *F*-values followed by *, **, and *** represent significance at *p* <0.05, *p* <0.01, and *p* <0.001, respectively; ns indicates no significant effect. BN207, Bainong 207; ZM136, Zhengmai 136; CK, water spray; N1, 5 mL/667 m^2^; N2, 10 mL/667 m^2^; N3, 15 mL/667 m^2^.

Compared with the CK treatment, BN207 under N2 exhibited significant increases of 58.5%, 57.4%, and 52.9% in grana number, grana lamellae number, and grana thickness, respectively. In ZM136, the corresponding increases were even higher, at 75.7%, 71.7%, and 64.3%, respectively (*p* < 0.05).

These results indicate that, under LT stress, the N2 treatment most effectively preserved the structural integrity of the thylakoid membrane system, thereby enhancing the light-harvesting capacity of the leaves. This conclusion is further supported by the ultrastructural changes observed using transmission electron microscopy.

### 3.3 Influence of the DA-6/28-HBL compound formulation on photosynthetic traits in wheat functional leaves under LT stress at the jointing stage

**Figure 4:**
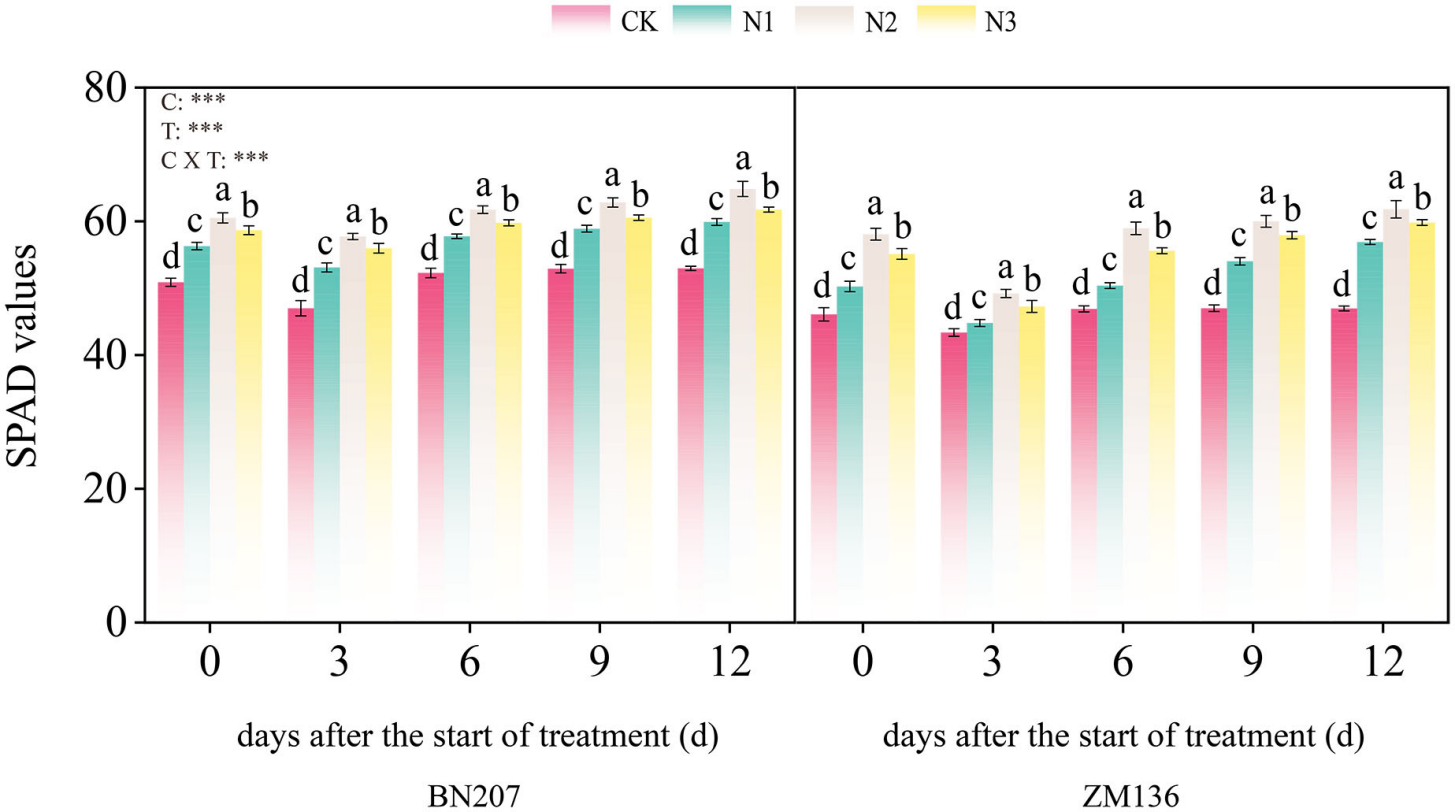
Effects of different concentrations of the DA-6/28-HBL compound formulation on SPAD values of functional leaves in two wheat cultivars. C, Cultivar; T, Treatment; BN207, Bainong 207; ZM136, Zhengmai 136; CK, water spray; N1, 5 mL/667 m^2^; N2, 10 mL/667 m^2^; N3, 15 mL/667 m^2^. Data are presented as mean ± SD (n = 3). Different lowercase letters indicate significant differences among treatments within each cultivar according to Duncan’s multiple range test at *p* < 0.05. *F*-values followed by *** indicate significance at *p* < 0.001. The two−way ANOVA results remained consistent across all time points.

The SPAD values of functional leaves in both cultivars decreased to their lowest levels 3 d after LT treatment (**Figure 4**). From 6 to 12 d after treatment, SPAD values in all treatments except CK gradually increased over time in both cultivars. In contrast, SPAD values in the CK treatment remained consistently low throughout this period. These results suggest that LT stress caused the most severe damage to functional leaves under the CK treatment, resulting in significantly lower SPAD values compared with the other treatments.

At the same time point, SPAD values of functional leaves in both cultivars showed a unimodal trend, initially increasing and then decreasing with increasing treatment concentration, with the highest values observed under the N2 treatment. Twelve days after treatment, SPAD values under N2 were significantly higher than those under CK, increasing by 22.4% in BN207 and 31.6% in ZM136 (*p* < 0.05). These findings demonstrate that the DA-6/28-HBL compound formulation effectively alleviated LT-induced damage to functional leaves and significantly improved SPAD values, with the N2 treatment showing the strongest effect. Additionally, although BN207 consistently exhibited higher overall SPAD values than ZM136, the latter showed greater responsiveness to the DA-6/28-HBL compound formulation.

**Figure 5:**
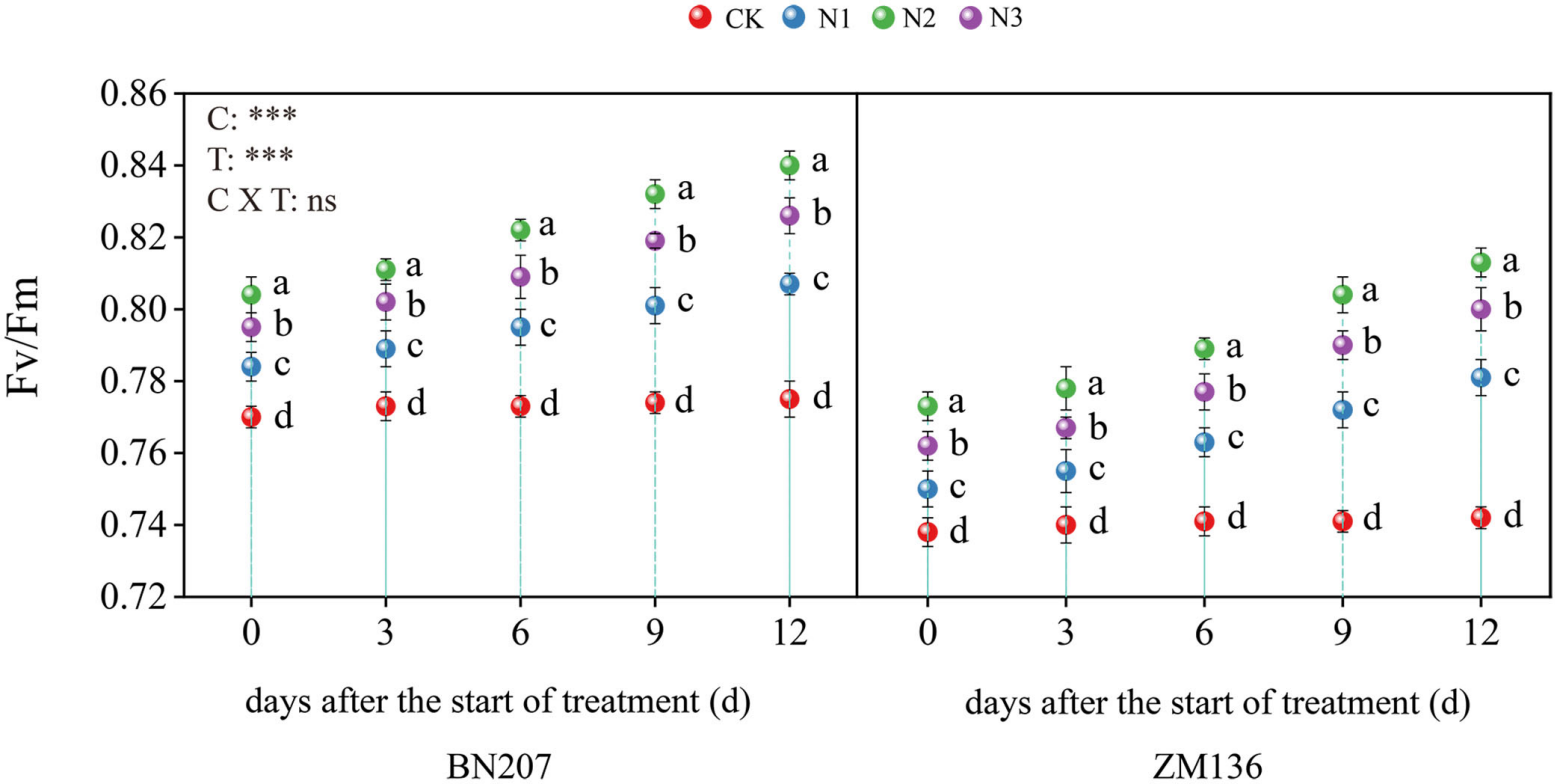
Effects of different concentrations of the DA-6/28-HBL compound formulation on the maximum photochemical efficiency (Fv/Fm) of functional leaves in two wheat cultivars. Data were obtained from dark-adapted leaves. C, Cultivar; T, Treatment; BN207, Bainong 207; ZM136, Zhengmai 136; CK, water spray; N1, 5 mL/667 m^2^; N2, 10 mL/667 m^2^; N3, 15 mL/667 m^2^. Data are presented as mean ± SD (n = 3). Different lowercase letters indicate significant differences among treatments within each cultivar according to Duncan’s multiple range test at *p* < 0.05. *F*-values followed by *** indicate significance at *p* < 0.001; ns indicates no significant effect. The two−way ANOVA results remained consistent across all time points.

**Figure 6:**
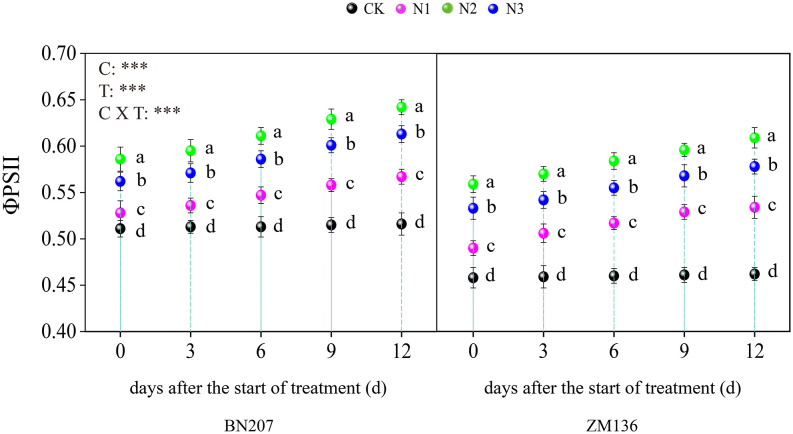
Effects of different concentrations of the DA-6/28-HBL compound formulation on the actual photochemical efficiency (*ΦPSII*) of functional leaves in two wheat cultivars. Data were obtained from light-adapted leaves. C, Cultivar; T, Treatment; BN207, Bainong 207; ZM136, Zhengmai 136; CK, water spray; N1, 5 mL/667 m^2^; N2, 10 mL/667 m^2^; N3, 15 mL/667 m^2^. Data are presented as mean ± SD (n = 3). Different lowercase letters indicate significant differences among treatments within each cultivar according to Duncan’s multiple range test at *p* < 0.05. *F*-values followed by *** indicate significance at *p* < 0.001. The two−way ANOVA results remained consistent across all time points.

As illustrated in **Figures 5** and **6**, compared with the CK treatment, application of the DA-6/28-HBL compound formulation consistently increased both Fv/Fm and *ΦPSII* in functional wheat leaves throughout the growth period, reaching peak values at 12 days after treatment.

At the same time point, both Fv/Fm and *ΦPSII* initially increased with increasing treatment concentration but declined at higher concentrations, reaching their highest values under the N2 treatment. Twelve days after treatment, Fv/Fm in BN207 and ZM136 under N2 increased significantly by 8.4% and 9.6%, respectively, compared with CK. Similarly, *ΦPSII* increased significantly by 24.4% and 31.8%, respectively (*p* < 0.05). These results indicate that the DA-6/28-HBL compound formulation significantly enhances both the maximum and actual photochemical efficiency of functional wheat leaves under LT stress. Moreover, this effect is concentration-dependent, with an optimal threshold observed at N2, which produced the most pronounced response.

### 3.4 Influence of the DA-6/28-HBL compound formulation on grain-setting rate and grain dry weight at different spikelet positions in wheat under LT stress at the jointing stage

**Figure 7:**
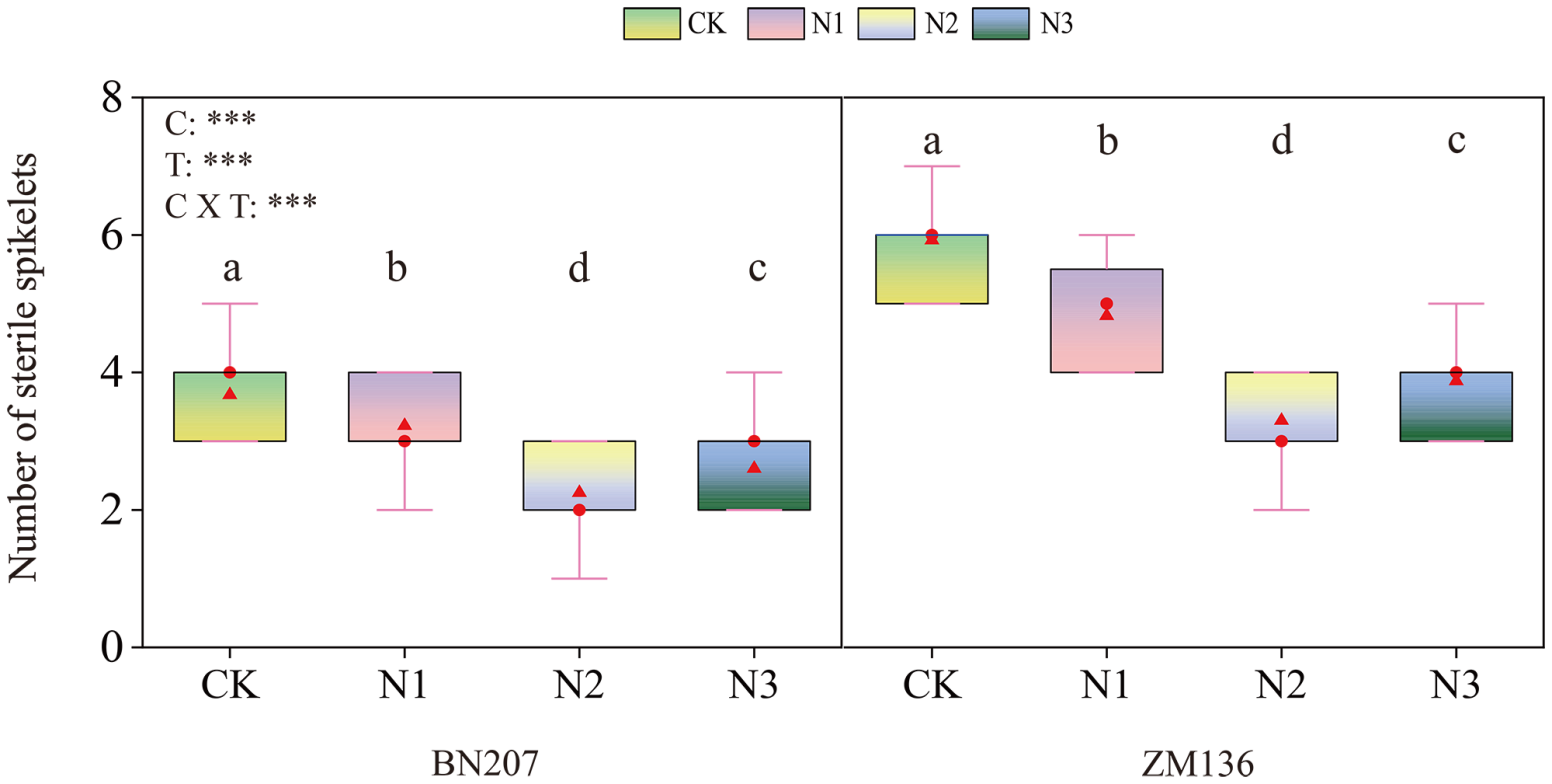
Effects of different concentrations of the DA-6/28-HBL compound formulation on the number of sterile spikelets in two wheat cultivars. Data are presented as box plots, with the boxes representing the interquartile range. Red triangles and circles indicate the mean and median, respectively. The upper and lower whiskers represent the maximum and minimum values, respectively. C, Cultivar; T, Treatment; BN207, Bainong 207; ZM136, Zhengmai 136; CK, water spray; N1, 5 mL/667 m^2^; N2, 10 mL/667 m^2^; N3, 15 mL/667 m^2^. Different lowercase letters indicate significant differences among treatments within each cultivar according to Duncan’s multiple range test at *p* < 0.05. *F*-values followed by *** indicate significance at *p* < 0.001.

As the concentration of the compound formulation increased, the number of sterile spikelets in both wheat cultivars initially decreased and then increased (**Figure 7**). Among all treatments, N2 resulted in the lowest number of sterile spikelets and differed significantly from all other treatments. Compared with the CK treatment, the number of sterile spikelets under N2 decreased significantly by 40.0% in BN207 and 44.3% in ZM136. These results indicate that application of the DA-6/28-HBL compound formulation at the N2 concentration prior to LT stress is the most effective strategy for reducing sterile spikelet number.

The wheat spikes were divided into three sections: upper, middle, and lower. For each section, the total number of set grains (**Supplementary Table 3**) and total grain weight (**Supplementary Table 4**) at different spikelet positions were recorded separately. Under LT stress, in both cultivars and across all treatments, the total number of set grains and total grain weight followed the same pattern across spike positions: middle section > upper section > lower section. Compared with plants that did not receive the DA-6/28-HBL compound formulation, foliar application of the formulation increased both the total number of set grains and total grain weight across all spikelet positions. As the treatment concentration increased, these parameters initially increased and then declined, reaching their maximum values under the N2 treatment, which differed significantly from the CK treatment. Specifically, compared with CK, the total number of set grains in BN207 under N2 increased significantly by 25.9%, 12.5%, and 5.8% in the upper, middle, and lower spikelet sections, respectively. In ZM136, the corresponding increases were 40.7%, 10.4%, and 6.3% (**Supplementary Table 3**). Regarding total grain weight at different spikelet positions under N2, BN207 showed significant increases of 45.0%, 17.7%, and 8.0% in the upper, middle, and lower sections, respectively, compared with CK. Similarly, ZM136 exhibited increases of 62.6%, 21.5%, and 10.0%, respectively (**Supplementary Table 4**).

A comparison of the total number of set grains and total grain weight at different grain positions within each spikelet section across treatments showed that, under the N2 treatment, BN207 exhibited significant increases compared with CK. Specifically, at the upper G2 position, the total number of set grains increased by 82.1% and total grain weight by 84.2%, while at the middle G4 position, the corresponding increases were 46.4% and 37.5%, respectively. For ZM136, significant increases were observed at the upper G1 position, with increases of 49.0% in total number of set grains and 73.0% in total grain weight, and at the middle G3 position, with increases of 33.3% and 51.7%, respectively (*p* < 0.05).

In summary, pre-treatment with the DA-6/28-HBL compound formulation prior to LT stress significantly increased both the total number of set grains and total grain weight across all spike positions, with the N2 concentration producing the strongest effect. In both wheat cultivars, upper spikelets showed greater increases in the total number of set grains and total grain weight than the middle and lower spikelets. The most pronounced improvements were observed at the upper G2 position in BN207 and the upper G1 position in ZM136.

### 3.5 Impact of the DA-6/28-HBL compound formulation on wheat grain yield and yield components under LT stress at the jointing stage

As shown in **Table 3**, compared with CK, all treatment concentrations significantly increased the number of grains per spike, 1000-grain weight, and grain yield per column in both wheat cultivars (*p* < 0.05). With increasing treatment concentration, these yield components initially increased and then decreased in both cultivars, reaching their maximum values under the N2 treatment. Further increases in concentration beyond N2 led to a significant decline in grain yield and its components. Specifically, compared with CK, N2 significantly increased the number of grains per spike by 13.7% and 14.2% in BN207 and ZM136, respectively, the 1000-grain weight by 5.9% and 9.3%, respectively, and the grain yield per column by 35.3% and 49.6%, respectively. These results indicate that foliar application of the DA-6/28-HBL compound formulation at the N2 concentration prior to LT stress most effectively enhanced grain yield, mainly by increasing both the number of grains per spike and 1000-grain weight.

**Table 3:** Effects of different concentrations of the DA-6/28-HBL compound formulation on grain yield and yield components in two wheat cultivars.

Cultivar	Treatment	Panicle number per plant	Kernel per spike	1000-kernel weight (g)	Yield per column (g)
BN207	CK	2.29 b	36.83 d	42.47 d	31.65 d
	N1	2.32 ab	38.86 c	43.40 c	35.06 c
	N2	2.34 a	41.89 a	44.98 a	42.81 a
	N3	2.33 a	40.97 b	44.25 b	40.11 b
ZM136	CK	2.16 b	34.44 d	43.89 d	26.76 d
	N1	2.18 ab	36.77 c	45.93 c	32.03 c
	N2	2.21 a	39.32 a	47.98 a	40.02 a
	N3	2.20 ab	38.55 b	47.57 b	38.29 b
F value	Cultivar	402.6***	51084.1***	25795.0***	44574.4***
	Treatment	12.1***	44593.9***	8396.0***	138735.6***
	Cultivar X Treatment	0.1ns	92.1***	673.1***	1869.8***

Data are presented as the mean (n = 3). Different lowercase letters indicate significant differences among treatments within each cultivar according to Duncan’s multiple range test at *p* < 0.05. *F*-values followed by *, **, and *** indicate significance at *p* < 0.05, *p* < 0.01, and *p* < 0.001, respectively; ns indicates no significant effect. BN207, Bainong 207; ZM136, Zhengmai 136; CK, water spray; N1, 5 mL/667 m^2^; N2, 10 mL/667 m^2^; N3, 15 mL/667 m^2^.

## 4 Discussion

### 4.1 Foliar application of the DA-6/28-HBL compound formulation prior to LT stress significantly influences leaf antioxidant activity and osmolyte accumulation in wheat

Under LT stress, excessive accumulation of ROS in plant cells triggers membrane lipid peroxidation, leading to increased MDA content and enhanced membrane permeability (Ozturk et al., 2020). To counteract oxidative damage, plants activate antioxidant enzyme systems to maintain ROS homeostasis. Among these enzymes, SOD scavenges superoxide anions, while CAT decomposes hydrogen peroxide (H_2_O_2_); their coordinated action effectively alleviates oxidative stress (Cannea and Padiglia, 2025). Previous research has shown that DA-6 treatment significantly increased SOD and CAT activities in kidney bean seedlings under chilling stress by 160.63% and 85.56%, respectively, after 24 h (Bai et al., 2025). Similarly, BRs have been reported to enhance cold tolerance in pepper by promoting antioxidant enzyme activity and accelerating ROS scavenging (Li et al., 2025). Building on this evidence, the present study investigated the regulatory effects of a DA-6/28-HBL compound formulation on LT tolerance in wheat. The results showed that foliar application of the DA-6/28-HBL compound formulation prior to jointing-stage LT stress significantly enhanced antioxidant enzyme activities and reduced MDA accumulation in both wheat cultivars, with a clear concentration-dependent effect. N2 (10 mL/667 m^2^) was identified as the optimal concentration under the experimental conditions. Specifically, compared with the CK treatment, SOD activity increased significantly by 90.6% and 94.1% in BN207 and ZM136, respectively, while CAT activity increased by 81.5% and 121.2%, respectively. In contrast, MDA content decreased significantly by 24.8% and 25.1%, respectively (**Figure 1**). These results are consistent with trends reported in previous studies. Previous mechanistic studies have shown that DA-6 mainly enhances cold tolerance by upregulating the ascorbate–glutathione (AsA–GSH) cycle and inducing the expression of CBF cold-responsive genes, thereby promoting ROS scavenging and cold acclimation (Lu et al., 2021; Bai et al., 2025). In contrast, BRs improve plant tolerance to LT stress through partially distinct pathways, including enhancement of glutathione S-transferase (GST) activity, maintenance of cellular redox homeostasis, and regulation of the alternative oxidase (AOX) pathway to preserve mitochondrial ROS balance (Shopova et al., 2021; Deng et al., 2015). These findings indicate that, although DA-6 and BRs act through distinct pathways, both enhance antioxidant capacity at the transcriptional and/or enzymatic level. This suggests that their combined application may exert synergistic effects on antioxidant enzyme activity through complementary mechanisms. However, whether the compound formulation acts through simultaneous activation of these pathways or involves additional regulatory nodes remains to be further investigated.

Osmolytes play a crucial role in plant stress responses by regulating osmotic potential and maintaining cellular structural and functional stability (Khan et al., 2023). Among them, soluble sugars, soluble proteins, and free proline are key osmolytes that help regulate osmotic balance in plant cells. Their accumulation adjusts cellular osmotic concentration, promotes abscisic acid (ABA) synthesis, induces stress-related protein synthesis, and enhances cold tolerance (Zhou et al., 2025). Previous studies have shown that exogenous DA-6 and BRs promote osmolyte accumulation through multiple regulatory pathways. DA-6 maintains increased ABA levels and induces CBF gene expression, thereby activating downstream cold-responsive genes and promoting the accumulation of compatible osmolytes, while also regulating endogenous cytokinin synthesis to stabilize chloroplast structure (Lu et al., 2021). BRs enhance proline accumulation by upregulating the activity and expression of key biosynthetic enzymes P5CS and P5CR while suppressing the activity of the catabolic enzyme PDH (Fu et al., 2019). They are also involved in regulating chloroplast gene expression and cellular water balance, thereby playing an important role in plant temperature adaptation (Sadura and Janeczko, 2022). Consistent with these findings, the present study demonstrated that exogenous foliar application of the DA-6/28-HBL compound formulation prior to LT stress significantly increased the contents of proline, soluble sugars, and soluble proteins in wheat leaves, with the most pronounced effects observed at 10 mL per 667 m^2^. These results indicate that the DA-6/28-HBL compound formulation may enhance wheat tolerance to LT stress by synergistically promoting osmolyte accumulation and maintaining osmotic homeostasis in leaf tissues. However, the specific transcriptional regulatory networks underlying the synergistic effects of the DA-6/28-HBL compound formulation remain unclear, and further investigation is required to elucidate its molecular mechanisms.

### 4.2 Foliar application of the DA-6/28-HBL compound formulation prior to LT stress enhances photosystem II function by stabilizing cellular structures

As the primary organ for photosynthesis, gas exchange, and environmental signal perception, leaf cellular ultrastructure serves as an important indicator of plant stress tolerance under adverse conditions (Yavas et al., 2024). Previous studies have reported that LT stress disrupts the regular arrangement of mesophyll cells in wheat leaves (Zhang et al., 2015). Consistent with these findings, the present study observed that, in the absence of the DA-6/28-HBL compound formulation, LT stress at the jointing stage caused substantial damage to leaf cellular ultrastructure, including disruption of mitochondrial structure, cytoplasmic vacuolization, and an increase in osmiophilic granules—an indicator of chloroplast senescence. Such structural damage directly contributes to a decline in photosynthetic performance (Domínguez and Cejudo, 2021). Among photosynthetic parameters, maximum photochemical efficiency (Fv/Fm) is a key indicator of photoinhibition and stress-induced injury. A decrease in Fv/Fm typically reflects inactivation or damage to the PSII reaction center, particularly the D1 protein, and the magnitude of this decline correlates positively with stress intensity (Guidi et al., 2019). Li et al. (2015) reported that exposure to low temperature (8 °C below ambient) during the jointing stage significantly reduced gas-exchange parameters and Fv/Fm in wheat leaves, resulting in a 5–14% reduction in grain yield. Consistently, the present study demonstrated that LT stress at the jointing stage significantly decreased Fv/Fm, PSII activity, and leaf SPAD values in wheat. Notably, SPAD values reached their minimum at the early stage of stress (3 d after LT treatment; **Figure 4**). This decline likely resulted not only from thylakoid membrane damage and inhibited chlorophyll biosynthesis but also from a photoprotective adaptive response, in which plants reduce pigment levels to limit excessive light absorption and mitigate photo-oxidative stress (Aslam et al., 2022; Guan et al., 2023).

As the primary site of photosynthesis, the structural organization of grana lamellae within chloroplasts directly influences leaf photosynthetic efficiency (Johnson and Wientjes, 2020). Previous studies have shown that exogenous growth regulators help maintain chloroplast integrity under stress conditions. For example, DA-6 treatment in tomato increased chloroplast number and grana lamellae abundance, inhibited chloroplast degradation, stabilized thylakoid membrane proteins, and consequently enhanced photosynthetic electron transport rate and photochemical activity (Lu et al., 2021). Similarly, seed soaking with exogenous BRs was reported to preserve the orderly arrangement of grana lamellae and the structural integrity of mitochondria, thereby improving PSII function and mitigating stress-induced damage (Salah et al., 2022). Building on these findings, the present study demonstrated that the combined application of DA-6 and BRs exerts a synergistic effect. Statistical analysis showed that, under LT stress, foliar application of the DA-6/28-HBL formulation significantly increased chloroplast area, grana number, grana lamellae count, and grana thickness in wheat functional leaves, thereby effectively maintaining the ordered stacking of the thylakoid membrane system compared with control plants. Transmission electron microscopy further confirmed that DA-6/28-HBL treatment preserved the regular elliptical shape of chloroplasts and increased their length-to-width ratio. Notably, the protective effect of DA-6 and 28-HBL on chloroplast ultrastructure is mainly mediated through an indirect pathway involving synergistic activation of the antioxidant defense system and alleviation of membrane lipid peroxidation. As shown in Section 4.1, DA-6 and 28-HBL synergistically enhanced the activities of antioxidant enzymes such as SOD and CAT, thereby effectively mitigating LT-induced lipid peroxidation damage. This reduction in oxidative damage provided a favorable membrane environment for the maintenance of chloroplast ultrastructure. These ultrastructural improvements enhanced the light-harvesting capacity of leaves, providing a cytological basis for the observed increases in SPAD values and PSII activity. Among the tested concentrations, application at 10 mL/667 m^2^ provided the most effective protection of cellular structures and the greatest improvement in PSII activity.

### 4.3 Foliar application of the DA-6/28-HBL compound formulation prior to LT stress improves grain set at different spikelet positions and yield components in wheat

Spatiotemporal heterogeneity in grain development occurs at different positions within the wheat spike, mainly due to uneven early differentiation of spike organs and asynchronous development of the vascular bundle system. This leads to spatial variation in grain set and dry matter accumulation across spike sections and individual grains (Yang et al., 2024). Previous studies have reported a “central dominance” pattern in wheat spike development, where grains from central spikelets exhibit higher fertility and greater grain weight, followed by those from the upper and lower spikelets (Luo et al., 2023). The findings of this study corroborate this pattern: under LT stress at the jointing stage, both wheat cultivars across all treatments showed a quadratic distribution pattern of fertile grain number and grain weight across spikelet and grain positions. This indicates that LT stress does not alter the inherent spatial distribution pattern of grain development within the spike.

Grain number per spike and 1000-grain weight are key determinants of wheat yield. This study found that, in the absence of the DA-6/28-HBL compound formulation, LT stress at the jointing stage significantly reduced both grain number per spike and 1000-grain weight, constituting the primary cause of yield loss. This reduction is largely attributed to LT stress accelerating floret degeneration, particularly in the upper florets, thereby impairing assimilate transport to spikelets, causing grain abortion and reducing grain set (Wang et al., 2009). In addition, LT stress reduces grain-filling potential by suppressing starch synthesis and accumulation, resulting in decreased grain weight (Ma et al., 2025). Consequently, the application of exogenous growth regulators to mitigate chilling injury and stabilize yield has become an important agronomic practice. For example, post-anthesis foliar application of DA-6 has been shown to increase 1000-grain weight by regulating the expression of key genes involved in protein and sucrose metabolism, thereby enhancing the synthesis of storage compounds (Xu et al., 2021a). Wang et al. (2024a) reported that precise regulation of the BRs signaling pathway using gene-editing technology, combined with stage-specific promoters to optimize BRs biosynthesis and distribution, can improve plant architecture, grain traits, and stress tolerance in wheat, ultimately enhancing yield. Similarly, Su et al. (2021) demonstrated that foliar application of monopotassium phosphate prior to late-spring cold events significantly reduced sucrose and fructose levels while increasing reducing sugar content in upper spikelets, thereby alleviating spikelet damage and improving the grain-setting rate.

This study examined the effects of combined DA-6 and 28-HBL application on wheat yield components under LT stress. The results demonstrate that foliar application of the DA-6/28-HBL compound formulation prior to LT stress mainly reduced yield losses by significantly increasing both grain number per spike and 1000-grain weight. Compared with CK, the N2 treatment (10 mL/667 m^2^) significantly increased grain yield per column by 35.3% in BN207 and 49.6% in ZM136, confirming 10 mL/667 m^2^ as the optimal concentration. By integrating chloroplast ultrastructure with antioxidant enzyme activities and osmotic adjustment substances, this study preliminarily elucidates the cold tolerance mechanism of the formulation from both physiological and structural perspectives. With a low application rate and simple operation, this formulation provides a feasible agronomic strategy for mitigating late−spring cold damage in the Huang−Huai region. However, higher concentrations may induce adverse effects, such as growth inhibition, hormonal imbalance, or potential environmental risks. Therefore, careful dose optimization is essential. Furthermore, these findings are based on a single-year column experiment conducted under simulated LT stress conditions. While this approach provides controlled and reproducible conditions for mechanistic investigation, it may not fully capture the complexity of natural field environments. Multi-year field trials across diverse climatic conditions are therefore necessary to validate the stability and reliability of the optimal application rate (10 mL/667 m^2^).

Further analysis of different spike positions revealed that the upper spike section showed the greatest increases in grain-setting rate and grain dry weight, significantly exceeding those in the middle and lower sections, consistent with previous findings. Additionally, at the individual grain position level, the most pronounced improvements occurred at the upper G2 position in BN207 and the upper G1 position in ZM136. This enhancement in grain setting at specific spikelet positions may be attributed to the synergistic regulatory effects of DA-6 and 28-HBL. As discussed in Sections 4.1 and 4.2, the compound formulation rapidly enhanced antioxidant enzyme activities and alleviated membrane lipid peroxidation, thereby preserving cellular structural integrity and promoting photosynthetic efficiency. Concurrently, the BR signaling pathway likely enhanced the transport and allocation of assimilates to sink organs, particularly to dominant grain positions (Han et al., 2023). These coordinated physiological responses synergistically mitigated yield loss under LT stress.

## 5 Conclusion

**Figure 8:**
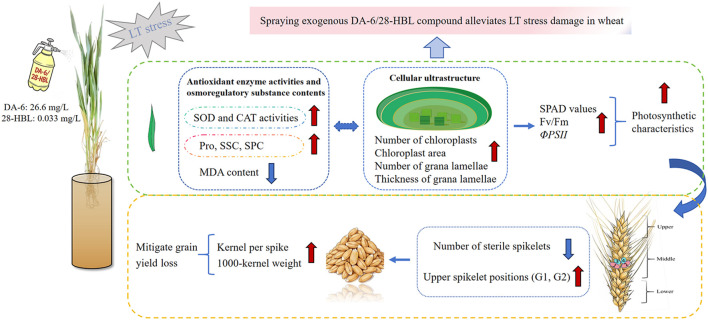
Foliar application of the DA-6/28-HBL compound formulation prior to LT stress enhances cold tolerance in wheat. Dashed arrows indicate indirect regulatory relationships. LT, low temperature; DA-6, diethyl aminoethyl hexanoate; 28-HBL, 28-homobrassinolide; SOD, superoxide dismutase; CAT, catalase; Pro, Proline; SSC, Soluble Sugar Content; SPC, Soluble Protein Content; MDA, malondialdehyde; Fv/Fm, maximum photochemical efficiency of PSII; *ΦPSII*, actual photochemical efficiency of PSII.

This study demonstrates that foliar application of the DA-6/28-HBL compound formulation effectively alleviated damage caused by LT stress at the wheat jointing stage, with 10 mL/667 m^2^ producing the most pronounced effect. Under LT stress, the treatment enhanced the activities of antioxidant enzymes, including SOD and CAT, in leaves while reducing MDA accumulation, thereby indirectly protecting chloroplast ultrastructure integrity. As a result, functional leaves maintained higher PSII activity and SPAD values, supporting the synthesis and accumulation of photosynthetic products. This ultimately led to significant improvements in grain-setting rate and grain weight at the upper spikelet positions (G1 and G2), increasing both grain number per spike and 1000-grain weight and thereby mitigating yield loss under LT stress (**Figure 8**). Among the two wheat cultivars tested, BN207 exhibited greater tolerance to LT stress.

## Data Availability

The original contributions presented in the study are included in the article/**Supplementary Material**. Further inquiries can be directed to the corresponding authors.
