# Supplementary material for: Foliar application of diethyl aminoethyl hexanoate and homobrassinolide enhances cold tolerance in wheat at the jointing stage: physiological mechanisms
Source: Front Plant Sci. 2026 Jul 27;17:1911607. doi: 10.3389/fpls.2026.1911607 (PMC13453770; doi:10.3389/fpls.2026.1911607)
Supplement: Supplementary file 1 [file SupplementaryFile1.docx]

**Supplementary Information**

**Legends**

**Supplementary table 1** Effects of different foliar spray treatments on wheat grain yield and yield components under low temperature stress at the jointing stage.

**Supplementary table 2** Cultivar characteristics.

**Supplementary figure 1** Rainfall distribution and the average temperature in the 2024–2025 wheat growing season.

**Supplementary table 3** Effects of different concentrations of the DA-6/28-HBL compound formulation on the total number of fertile grains at different spikelet positions in two wheat cultivars.

**Supplementary table 4** Effects of different concentrations of the DA-6/28-HBL compound formulation on grain dry weight at different spikelet positions in two wheat cultivars.

**Supplementary table 1.** Effects of different foliar spray treatments on wheat grain yield and yield components under LT stress at the jointing stage.

| **Cultivars** | **Treatment** | **Panicle number**  **per plant** | **Kernel per**  **spike** | 1000-**kernel**   **weight (g)** | **Yield per**  **column (g)** |
| --- | --- | --- | --- | --- | --- |
|  |  |  |  |  |  |
|  | CK | 2.29 b | 37.00 c | 42.53 c | 32.08 d |
|  | 28-HBL | 2.30 ab | 38.44 b | 43.16 b | 34.57 c |
| BN207 | DA-6 | 2.33 ab | 39.10 b | 43.58 b | 35.73 b |
|  | DA-6/28-HBL | 2.36 a | 42.00 a | 45.33 a | 43.08 a |
|  | CK | 2.17 b | 34.57 c | 43.95 d | 27.11 d |
|  | 28-HBL | 2.18 b | 36.43 b | 45.66 c | 31.62 c |
| ZM136 | DA-6 | 2.19 ab | 37.29 b | 46.12 b | 32.89 b |
|  | DA-6/28-HBL | 2.23 a | 39.43 a | 48.03 a | 40.67 a |
|  | Cultivar | 107.9*** | 106.5*** | 612.7*** | 6008.6*** |
| *F* value | Treatment | 5.5** | 92.0*** | 238.8*** | 14833.6*** |
|  | Cultivar X Treatment | 0.2ns | 0.7ns | 10.1*** | 180.7*** |

Data presented in the table are from the column cultivation experiment conducted during the 2023–2024 growing season. LT, low temperature; BN207, Bainong 207; ZM136, Zhengmai 136; CK, water spray; DA-6, diethyl aminoethyl hexanoate alone at a concentration of 26.6 mg/L; 28-HBL, 28-homobrassinolide alone at a concentration of 0.033 mg/L; DA-6/28-HBL, combined foliar spray of 26.6 mg/L diethyl aminoethyl hexanoate and 0.033 mg/L 28-homobrassinolide. Data are presented as the mean (n = 3). Different lowercase letters indicate significant differences among treatments within each cultivar according to Duncan's multiple range test at *p* < 0.05. *F*-values followed by *, **, and *** indicate significance at *p* < 0.05, *p* < 0.01, and *p* < 0.001, respectively; ns indicates no significant effect.

**Supplementary table 2.** Cultivar characteristics.

| **Cultivars** | **Maternal parent** | **Paternal parent** | **Tolerance to late-spring coldness** |
| --- | --- | --- | --- |
| Bainong 207 | Zhoumai 16 | Bainong 64 | Relatively strong |
| Zhengmai 136 | Aikang 58 | Jimai 22 | Moderate |

**Supplementary figure 1.** Monthly average rainfall and temperature during the wheat growth period in 2024–2025.

**Supplementary table 3.** Effects of different concentrations of the DA-6/28-HBL compound formulation on the total number of fertile grains at different spikelet positions in two wheat cultivars.

| **Cultivars** | **Treatment** | **Upper** | | **Middle** | | | | **Lower** | | |
| --- | --- | --- | --- | --- | --- | --- | --- | --- | --- | --- |
|  |  | **G1** | **G2** | **G1** | **G2** | **G3** | **G4** | **G1** | **G2** | **G3** |
|  | CK | 4.34 d | 1.45 d | 9.49 d | 8.91 d | 5.79 d | 0.84 c | 3.31 c | 1.91 c | 0.82 b |
|  | N1 | 4.43 c | 2.04 c | 9.61 c | 9.05 c | 6.88 c | 1.07 b | 3.36 bc | 1.95 bc | 0.84 b |
| BN207 | N2 | 4.65 a | 2.64 a | 9.87 a | 9.27 a | 7.80 a | 1.23 a | 3.47 a | 2.03 a | 0.88 a |
|  | N3 | 4.59 b | 2.52 b | 9.81 b | 9.18 b | 7.52 b | 1.19 a | 3.43 ab | 1.99 b | 0.87 a |
|  | CK | 3.92 d | 0.99 c | 9.25 d | 8.62 d | 5.22 d | 0.76 c | 3.16 b | 1.79 c | 0.74 c |
|  | N1 | 4.83 c | 1.03 bc | 9.36 c | 8.69 c | 6.05 c | 0.85 b | 3.23 ab | 1.85 b | 0.76 b |
| ZM136 | N2 | 5.84 a | 1.07 a | 9.58 a | 8.83 a | 6.96 a | 0.95 a | 3.32 a | 1.93 a | 0.80 a |
|  | N3 | 5.66 b | 1.05 ab | 9.53 b | 8.80 b | 6.76 b | 0.93 a | 3.29 a | 1.90 ab | 0.78 b |
|  | Cultivar | 2733.9^***^ | 7087.3^***^ | 1345.3^***^ | 2122.6^***^ | 3116.9^***^ | 772.6^***^ | 103.3^***^ | 118.5^***^ | 378.1^***^ |
| *F* value | Treatment | 2264.9^***^ | 463.6^***^ | 515.4^***^ | 251.5^***^ | 3902.1^***^ | 301.7^***^ | 25.3^***^ | 40.2^***^ | 44.7^***^ |
|  | Cultivar X Treatment | 1191.0^***^ | 360.9^***^ | 2.0ns | 14.1^***^ | 21.7^***^ | 35.9^***^ | 0.2ns | 0.6ns | 0.4ns |

Spikelets were grouped into three sections: lower (basal six spikelets), upper (apical six spikelets), and middle (remaining spikelets). G1, G2, G3, and G4 denote grains at the 1ˢᵗ, 2ⁿᵈ, 3ʳᵈ, and 4ᵗʰ positions within a spikelet, respectively. BN207, Bainong 207; ZM136, Zhengmai 136; CK, water spray; N1, 5 mL/667 m²; N2, 10 mL/667 m²; N3, 15 mL/667 m². Data are presented as the mean (n = 3). Different lowercase letters indicate significant differences among treatments within each cultivar according to Duncan's multiple range test at *p* < 0.05. *F*-values followed by *, **, and *** indicate significance at *p* < 0.05, *p* < 0.01, and *p* < 0.001, respectively; ns indicates no significant effect.

**Supplementary table 4.** Effects of different concentrations of the DA-6/28-HBL compound formulation on grain dry weight at different spikelet positions in two wheat cultivars.

| **Cultivars** | **Treatment** | **Upper** | | **Middle** | | | | **Lower** | | |
| --- | --- | --- | --- | --- | --- | --- | --- | --- | --- | --- |
|  |  | **G1** | **G2** | **G1** | **G2** | **G3** | **G4** | **G1** | **G2** | **G3** |
|  | CK | 134.7 d | 54.3 d | 468.0 d | 443.2 d | 218.6 d | 37.6 d | 137.6 c | 48.4 c | 31.8 c |
|  | N1 | 153.7 c | 76.2 c | 490.9 c | 460.9 c | 266.4 c | 44.6 c | 141.0 b | 50.4 b | 33.2 b |
| BN207 | N2 | 174.0 a | 100.0 a | 517.6 a | 485.9 a | 318.3 a | 51.7 a | 147.2 a | 53.1 a | 35.0 a |
|  | N3 | 168.2 b | 93.1 b | 511.6 b | 479.2 b | 304.5 b | 49.8 b | 145.8 a | 52.4 a | 34.6 a |
|  | CK | 115.0 d | 47.9 d | 474.7 d | 437.3 d | 198.6 d | 47.2 d | 126.4 d | 48.1 c | 28.2 b |
|  | N1 | 155.4 c | 55.7 c | 498.8 c | 469.9 c | 247.4 c | 57.6 c | 130.9 c | 50.5 b | 29.3 b |
| ZM136 | N2 | 198.9 a | 66.0 a | 528.4 a | 506.8 a | 301.3 a | 70.4 a | 138.8 a | 52.9 a | 31.2 a |
|  | N3 | 183.8 b | 62.4 b | 522.4 b | 500.2 b | 287.5 b | 65.9 b | 136.7 b | 52.3 a | 30.8 a |
|  | Cultivar | 116.7^***^ | 5019.8^***^ | 578.9^***^ | 689.9^***^ | 1930.8^***^ | 3561.7^***^ | 1625.9^***^ | 0.4ns | 432.2^***^ |
| *F* value | Treatment | 2763.5^***^ | 1916.4^***^ | 3917.5^***^ | 3558.7^***^ | 11961.9^***^ | 1171.2^***^ | 433.3^***^ | 153.5^***^ | 60.8^***^ |
|  | Cultivar X Treatment | 351.1^***^ | 366.9^***^ | 7.6^***^ | 222.3^***^ | 3.2^*^ | 66.1^***^ | 6.6^**^ | 0.4ns | 0.2ns |

mg. Spikelets were grouped into three sections: lower (basal six spikelets), upper (apical six spikelets), and middle (remaining spikelets). G1, G2, G3, and G4 denote grains at the 1ˢᵗ, 2ⁿᵈ, 3ʳᵈ, and 4ᵗʰ positions within a spikelet, respectively. BN207, Bainong 207; ZM136, Zhengmai 136; CK, water spray; N1, 5 mL/667 m²; N2, 10 mL/667 m²; N3, 15 mL/667 m². Data are presented as the mean (n = 3). Different lowercase letters indicate significant differences among treatments within each cultivar according to Duncan's multiple range test at *p* < 0.05. *F*-values followed by *, **, and *** indicate significance at *p* < 0.05, *p* < 0.01, and *p* < 0.001, respectively; ns indicates no significant effect.
